# The local environment influences salt tolerance differently in four *Salicornia europaea* L. inland populations

**DOI:** 10.1038/s41598-025-97394-5

**Published:** 2025-04-16

**Authors:** Aleksandra Orzoł, Katarzyna Głowacka, Ricarda Pätsch, Agnieszka Piernik, Susana Dianey Gallegos-Cerda, Stefany Cárdenas-Pérez

**Affiliations:** 1 Department of Geobotany and Landscape Planning, Faculty of Biological and Veterinary Sciences, Nicolaus Copernicus University in Toruń, 87-100 Toruń, Poland; 2 Department of Plant Physiology, Genetics and Biotechnology, University of Warmia and Mazury in Olsztyn, Oczapowskiego 1a, 10-719 Olsztyn, Poland; 3 IBU Institute of Biology and Environmental Science, Carl Von Ossietzky Universität Oldenburg, 26129 Oldenburg, Germany; 4 Department of Botany and Zoology, Faculty of Science, Masaryk University, Brno, Czech Republic

**Keywords:** Enzyme activity, Halophyte, Plant-soil relationship, Salinity adaptation, Salt-affected environments, Salt-stress biomarkers, Ecology, Physiology, Plant sciences, Ecology, Environmental sciences

## Abstract

Salinity limits plant growth and crop production, impacting 8.7% of the earth’s surface. Plants growing in saline soils have adaptations that help them persist in these harsh environments. In this research, we studied the salt-stress response mechanism of four populations of *Salicornia europaea* by varying the salinity gradient between 0 and 1000 mM. Our results demonstrate that salinity changes the morphological traits, salinity stress biomarkers, and the activity of antioxidative enzymes in the shoots and roots of these plants differently. The present results suggest that plants from the Salzgraben Salzdahlum population in Germany were the most tolerant to salinity, followed by Inowrocław in Poland, which exhibited a higher content of CAT in roots at 1000 mM, which we attributed to its higher salt tolerance. The differential behavior in *Salicornia* populations confirms that the tolerance mechanism is population-specific. This study is essential for advancing saline agriculture, developing restoration strategies for saline areas, and exploring *S. europaea* as a potential functional food. The strong association between halophyte salinity tolerance, high biomass production, and enhanced cellular antioxidant defenses highlights its resilience and suitability for these applications.

## Introduction

Salinity is one of the main factors affecting plant growth and crop production. High soil salinity significantly threatens modern agriculture, exacerbated by land use and climate change^1^. Inappropriate water management, irrigation with increasingly saline water, reduced rainfall, increased temperature and evapotranspiration contribute to increasing salinity in arid and semi-arid regions^2^. Today, saline soils cover more than 833 million hectares worldwide, 8.7% of the earth’s surface^3^.

Food and Agriculture Organization (FAO) declared that soil salinity causes up to 1.5 million hectares of farmland to be withdrawn from production each year^3^. Saline soils develop through the accumulation of water-soluble calcium, magnesium, potassium, and sodium salts, causing fast-dwindling freshwater sources and reducing soil fertility^4,5^.The limit for soil salinity in crops depends on the specific species and its tolerance to salt stress.

Plants growing in saline soil have several meaningful responses due to the challenging conditions for development^6^. Most research has shown that salinity primarily induces osmotic stress (i.e., physiological drought) and water deprivation^7,8^. High soil salinity levels cause low germination rates and reduced crop growth and yield of crops^6^. In response to soil salinity, plants have evolved physiological and biochemical mechanisms to adapt and tolerate salt stress^9^. This action involves the biochemical pathways of ion homeostasis, osmolyte synthesis, removal of reactive oxygen species (ROS), and plant hormonal balance^7^. However, some plant species, namely halophytes, can tolerate much higher soil salinity levels than others^10,11^. Some halophytes properly tolerate stress factors since their natural habitats are often affected by floods, droughts, and high temperatures^12^.

According to soil classification, the saline soils have an electrical conductivity (ECe) of more than four dS m^−1^ at 25 °C (equivalent to < 40 mM NaCl)^13^. However, the salinity threshold tolerance for most vegetable crops is relatively low (from 1 to 2.5 dS m^−1^)^14^. Species of the genus *Salicornia* are among the plants with the highest productivity and salinity tolerance among halophytes^11,15,16^.

To determine the tolerance of crop plants to salinity, researchers examine the reduction in biomass yield from saline soils compared to yields from non-saline soil^17,18^. The latest studies have reported *S. europaea*'s optimum salinity for biomass production is between 200 and 400 mM NaCl under laboratory conditions^19^ and in situ in temperate inland salt marshes, i.e. 38.1 dS m^−1^^20^. Therefore, plant growth of this species can be negatively affected by the ranges below or above the limit values^19,21,22^.

*Salicornia* spp. is used for food and fodder purposes^23^ and production of biofuels^24^. Due to its high salt tolerance, *S. europaea* is recognized as a model plant for studying the molecular mechanisms of halophytes under salinity^10^. Salinity promotes the growth of this species by increasing tissue succulence and maintaining relatively stable K^+^ concentrations^10,19^. Their resistance to abiotic stress is due to genetic conditioning because these plants carry a gene from the family xyloglucan endotransglycosylase/hydrolase^25,26^. Moreover, their higher salt tolerance is also linked to changes in anatomical and biochemical traits, including oxidative stress biomarkers^21,27^.

The physiological, biochemical, and molecular mechanisms behind the tolerance and adaptation of these plants to salinity stress are not yet fully understood. It is already known that the reactive oxygen species (ROS) at moderate levels are beneficial, acting as key messengers, supporting signaling and plant development^28–30^, but an overproduction of them is the most frequent phenomenon during plant salt stress, which activates the antioxidant defence systems^31^. During this process, antioxidant enzymes are crucial in mitigating oxidative damage in the plant cells^2^.

Few researchers have studied the growth dynamics and salinity tolerance of different populations within the same species. Ibraheem et al.^32^ studied *Suaeda monoica Forssk. ex J.F.Gmel., Suaeda vermiculata Forssk. ex J.F.Gmel.*, and *Suaeda schimperi* Moq. against abiotic stress in their natural saline environment. They found that specific tolerance mechanisms are driven by the level of salinity and the genetic constitution of *Suaeda* species. More recently, research on two *S. europaea* populations found that the population with higher salt tolerance may have adapted due to a less stressed maternal environment, enhancing its adaptive plasticity under severe salinity^18,33^. Studies on the salinity tolerance of *S. europaea* are typically conducted using populations geographically close to the research group^34^. However, the findings and mechanisms of adaptation to salinity are often generalized to this species without accounting for the fact that different populations within the same species may function as distinct ecotypes^23^. These ecotypes can exhibit varying levels of salinity tolerance depending on their maternal-environmental conditions.

Therefore, it is vital to determine how salinity affects the growth of *S. europaea* and the mechanisms that underlie these effects depending on their environment. *S. europaea* adjusts its morphological traits and biochemical processes when subjected to different salinity levels. However, it remains unclear whether different populations of the same species display distinct salinity tolerance and responses when exposed to varying salinity levels.

*Salicornia* “adaptive strategies” may differ depending on the plant’s organs (shoots and roots) and even within populations. The plant’s diverse salt stress adaptation strategies, both above and below ground, are vital for advancing saline agriculture. They provide valuable insights into optimal agronomic conditions for its successful cultivation^7^. We tested the hypothesis that under controlled conditions, the local environment shapes populations’ salt-stress responses differently by assessing the morphological and biochemical effects in shoots and roots of four geographically distinct inland *S. europaea* populations. Our study aims to investigate the effects of salinity on morphology and the changes occured in the selected stress biomarkers (hydrogen peroxide [H_2_O_2_], malondialdehyde [MDA], and proline [PROL]), and key antioxidant enzymes (superoxide dismutase [SOD], peroxidase [POD], and catalase [CAT]) across four inland populations to gain insights into their performance under saline conditions.

## Materials and methods

### Methods

We collected *S. europaea* seeds from four inland populations (two in Germany, and two in Poland). They were grown under controlled conditions with varying salinity levels (0, 200, 400, 600, 800, and 1000 mM). Following two months of complete development of *S. europaea*, we measured shoot and root traits: length, biomass, water content, salinity stress biomarkers: hydrogen peroxide (H_2_O_2_), malondialdehyde (MDA), and proline (PROL), and antioxidant enzyme activity: guaiacol peroxidase (POD), catalase (CAT), and superoxide dismutase (SOD). Statistical methods were used, including two-way ANOVA, principal component analysis (PCA), and Non-metric multidimensional scaling (NMDS), to analyze variations among the salinity responses within populations and salt treatments.

### Experiment design

We collected *S. europaea* seeds from four spatially separated inland populations representing four different ecotypes: Inowrocław (*Inow*), (52° 48′ N, 18° 15′ E), Ciechocinek (*Ciech*), in Poland (52° 53′ N, 18°47′ E), Salzgraben Salzdahlum (*Salz*), (52° 11′ 56.9′′ N, 10° 36′ 05.0′′ E) and Soltauquelle (*Soltq*) in Germany (52° 05′ 24.2′′ N, 10° 49′ 18.2′′ E). All methods were performed in accordance with the relevant guidelines, regulations and legislation. We obtained permissions from Poland (Regional Director of Environmental Protection in Bydgoszcz, WOP.6400.12.2020. JC) and Germany (Untere Naturschutz- und Waldbehörde Helmstedt and the Natur- und Landschaftsschutz, Landkreis Wolfenbüttel), which we will provide per request. Dr. Agnieszka Piernik and Dr. Ricarda Pätsch formally identified plant species in Poland and Germany respectively. The voucher specimen of the plant material has been deposited in the publicly available herbarium of the Nicolaus Copernicus University in Torun (Index Herbarium code TRN). The deposition number is not available.

*Salicornia europaea* seeds were germinated in three Petri dishes (⌀ 18 cm) per population on filter paper moistened with distilled water. Once seedlings emerged, we prepared 18 plastic pots (5.5 × 5.3 cm, ~ 125 cm^3^) for each salinity treatment, using a 1:1 mixture of vermiculite and sand as the substrate.

Before planting, each set of 18 pots was placed on individual trays without drainage and fully saturated with 0, 200, 400, 600, 800, or 1000 mM NaCl solutions. To ensure uniform salinity, 600 mL of the prepared solution was added per 12 pots, allowing full substrate saturation without standing water. A single seedling was then planted per pot following the design of Cárdenas-Pérez et al.^33^.

For the first 30 days, seedlings were watered with distilled water as needed to maintain moisture and prevent dehydration. Thereafter, to ensure consistent hydration and nutrient availability, each tray received 250 mL of Hoagland’s solution (pH 7) every 1–2 days as required. We continuously monitored salinity levels in the trays using a salinity refractometer.

The germination and growth of plants were carried out under controlled conditions in a growth chamber under 50–60% relative humidity at 25/20 °C, in the 16/8 h day-to-night ratio and photon flux density of 1000 mmol m^−2^ s^−1^.

**Fig. 1:**
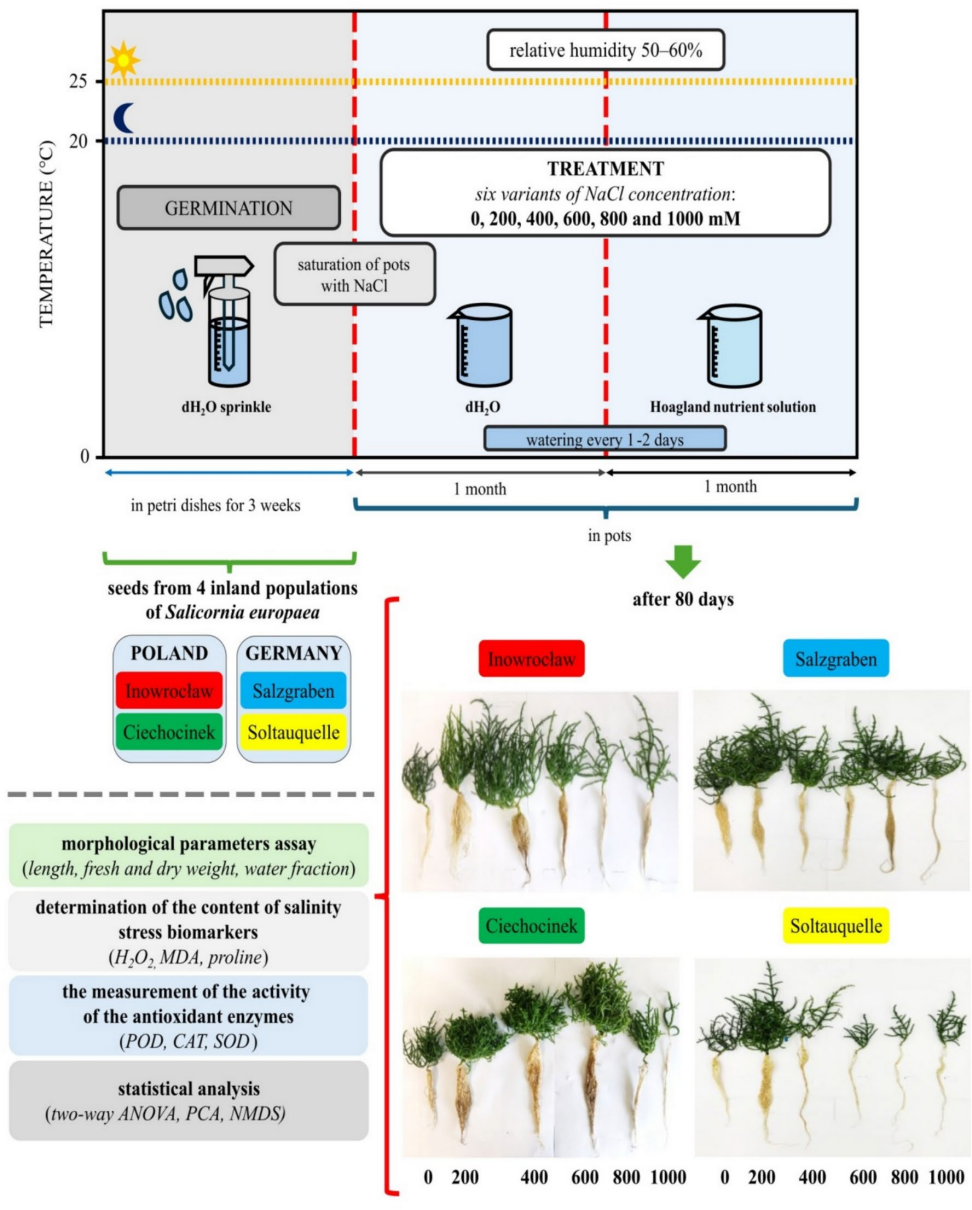
Graphical design of the experimental study on *S. europaea* showing the process from germination through cultivation conditions, including the tested salt-stress biomarkers and subsequent analyses. Photographs of the four populations are included, highlighting shoots and roots for comparison.

After 80 days of plant development, the growth and biochemical parameters of all plants were evaluated (Fig. 1). Morphological parameters, including length, fresh weight, dry weight, and water fraction, were assessed first. Subsequently, the levels of the selected salinity stress biomarkers—H_2_O_2_, MDA, and proline—were measured, alongside the activity of antioxidant enzymes (guaiacol peroxidase [POD], catalase [CAT], and superoxide dismutase [SOD]) in both shoots and roots. All plants were randomly selected for analysis, with five plants chosen for morphometric evaluation and three plants per population designated for biochemical analysis.

### Plant growth analysis

The lengths of shoots and roots (S-Length and R-Length, respectively) were measured (n = 5) using images captured from the plants’ shoots and roots. The imaging setup included a photography light box (PULUZ, PU5060, HITSAN, China) and a Fujifilm FinePix S2000HD digital camera (10 MP, f/3.5, 1/2.3", focal length 3.79 mm, with autofocus). Following previous protocols^27^, measurements were performed using the ImageJ software (version 1.53k). The fresh weight (FW) of shoots and roots was determined, and dry weight (DW) was measured after drying the samples at 85 °C for 72 h (S-FW and R-FW, S-DW and R-DW, respectively). The water content fraction of shoots and roots (S-WF and R-WF) was calculated using the formula: (FW–DW)/FW.

### Determination of salinity stress signalling biomarkers

200 mg FW samples were taken from both shoots and roots for stress biomarker analysis and enzyme activity measurements. Samples were randomly selected, and triplicates were considered for each analysis.

#### Evaluation of hydrogen peroxide content

The hydrogen peroxide content in shoots and roots (S-H_2_O_2_ and R-H_2_O_2_) were determined according to Junglee et al.^35^ methods. Shoot and root samples were homogenized with 1 mL of buffer containing 0.25 mL 0.1% (w:v) trichloroacetic acid, 0.5 mL 1 M KI and 0.25 mL 10 mM of potassium phosphate buffer solution (pH = 7.8) on an ice bath for 10 min. The homogenate was centrifuged for 15 min at 12,000 × *g* at 4 °C. The supernatants were collected; 200 μL of each supernatant were placed in UV-microplate wells and left to incubate at room temperature for 20 min. The absorbance was measured with a spectrophotometer (Infinite® 200 PRO NanoQuant, Tecan) at λ = 390 nm. The H_2_O_2_ content was determined on a standard curve from 0 to 40 mM. The H_2_O_2_ content was expressed in nM mg^−1^ FW.

#### Determination of malondialdehyde content

Malondialdehyde (MDA) content of shoots and roots (S-MDA and R-MDA) was determined according to Dhindsa et al.^36^. Shoots and roots were homogenized in an ice bath on a 0.1% trichloroacetic acid (TCA). The homogenates were centrifuged for 5 min at 10,000 × *g* at 4 °C and supernatants were collected for analysis. The reaction mixture consisted of 400 μL of 0.5% thiobarbituric acid (TBA) prepared in 20% TCA. The supernatant (250 μL) was added to the reaction mixture and incubated at 95 °C for 30 min in a termoblock (Termoblock Bio TDB-100, BioSan). The sample was cooled on ice for 5 min, and the absorbance was measured with a spectrophotometer (Cecil Instruments, CE 2501) at λ = 532 nm and λ = 600 nm (non-specific absorbance) against a blank without extract. MDA concentrations were determined using the extinction coefficient 155 mM^−1^ × cm^−1^. The MDA content was expressed in mM mg^−1^ FW.

#### Determination of proline content

The proline content of shoots and roots (S-PROL and R-PROL) was measured according to Chen and Zhang^37^. On ice, shoots and roots were homogenized in 3 mL of 100 mM PBS buffer (pH = 7.8) for 5 min. The homogenates were centrifuged for 20 min at 10,000 × *g* at 4 °C, and then supernatants were carefully collected. 50 μL of supernatant was added to the 1 mL reaction solution containing 10 mL of 3% sulphosalicylic acid, 10 mL of acetic acid, and 20 mL of 2.5% acid-ninhydrin. The samples were boiled in a water bath for 15 min and then cooled on an ice bath for 5 min. The absorbance was measured with a spectrophotometer (Infinite® 200 PRO NanoQuant, Tecan) at λ = 520 nm against a blank with 50 μL 100 mM PBS (pH 7.8). The L-proline curve (from 0 to 30 µg) was used to determine the proline content in the samples, which was expressed in µg g^−1^ fresh weight.

### Activity of antioxidant enzymes

#### Guaiacol peroxidase activity assay

Plant extracts were prepared in an ice bath according to Ziółkowska et al.^38^. FW of shoots and roots were homogenized for 30 min in a buffer solution containing 0.1 M Tris–HCl, 8.75% PVP, 0.1 M KCl, and 0.28% Triton X-100. The samples were centrifuged for 30 min at 4000 × *g* at 4 °C. Protein in the samples was analyzed by the Bradford method^39^. The activity of guaiacol POD of shoots and roots (S-POD and R-POD) was determined spectrophotometrically (Cecil Instruments, CE 2501) in the reaction mixture containing 80 µL 1% guaiacol, 1.5 mL 0.1 M KH_2_PO_4_ and 10 µL 0.06% H_2_O_2_. The absorbance growth rate was measured at room temperature at the wavelength of λ = 470 nm. One unit (U) corresponds to the oxidation of 1 μM H_2_O_2_ for 1 min mg^−1^ protein g^−1^ FW.

#### Catalase activity assay

Plant extracts were prepared according to Aebi^40^. FW of shoots and roots were homogenized in 50 mM phosphate buffer solution (pH = 7), which contained 10 g L^−1^ PVP, 0.2 mM EDTA and 10 mL L^−1^ Triton X-100. The samples were centrifuged for 20 min at 12,000 × *g* at 4 °C. The supernatant was separated from the sediment. The proteins in the samples were analyzed using the Bradford method. The activity of catalase (CAT) of shoots and roots (S-CAT and R-CAT) was determined spectrophotometrically (Cecil Instruments, CE 2501) in a reaction mixture containing 50 mM phosphate buffer (pH = 7) and 15 mM H_2_O_2_. The absorbance was measured for 10 min at room temperature at the wavelength of λ = 240 nm. One unit (U) corresponds to the oxidation of 1 μM H_2_O_2_ for 1 min mg^−1^ protein g^−1^ FW.

#### Superoxide dismutase activity assay

Plant extracts were prepared according to the method proposed by Alici and Arabaci^41^. Shoots and roots were homogenized in 100 mM PBS buffer solution (pH = 7) containing one mM ascorbic acid and 0.5% PVP for 5 min. The samples were centrifuged for 15 min at 5000 × *g* at 4 °C. The supernatant was separated from the sediment. Then, the Bradford method protein was assayed in the samples. The SOD activity of shoots and roots (S-SOD and R-SOD) was determined according to the method described by Zhang et al.^42^ with some modifications. For activity analysis, 60 μL of supernatant was added to 130 μL of reaction buffer containing 1.5 M sodium carbonate, 200 mM methionine, 2.25 mM NBT, three mM EDTA and 100 mM PBS (pH = 7.5). After mixing, 60 μM riboflavin was added. Samples were incubated in UV-microplate wells under light intensity of 4000 l× for 15 min. The absorbance was measured at a wavelength of λ = 560 nm in the dark (Infinite® 200 PRO NanoQuant, Tecan). One unit of SOD was defined as the amount of enzyme that inhibits 50% nitroblue tetrazolium photoreduction and was expressed as mg^−1^ protein g^−1^ FW.

### Statistical analysis

Population’s growth performance, oxidative stress biomarkers and antioxidative stress defence were compared through a two-way ANOVA with the Holm-Sidak method *p* < 0.05, SigmaPlot v. 14.0 (SigmaPlot v. 14). The significance of main effects between the four populations, between salinity treatments and their interactions were considered.

PCA was performed to relate biochemical responses to the growth performance of *S. europaea* populations. It was done twice for shoot and root biochemical parameters, correlating them to the morphological parameter

The population’s performance along the stress gradient was compared using NMDS with Bray–Curtis dissimilarity based on shoot morphological parameters and biochemical responses. This analysis was performed to compare populations based on very complex data and to identify the distance between them in the ordination space. Treatments non-salinity (0 mM), optimum range (200–400 mM) and severe salt stress (1000 mM) were included in both analyses. The PCA was performed in XLSTAT (XLSTAT, 2023), and the non-metric multidimensional scaling in PAST 4.09.

## Results

Our analysis demonstrated that *S. europaea* populations, salinity levels and their interaction significantly influenced most of the studied parameters. Optimal growth occurred at salinity levels of 200–400 mM, with the highest growth at the upper end. Stress biomarkers, such as H_2_O_2_ and MDA, peaked in plants without NaCl and at 1000 mM. The absence of salinity or exposure to a severe salt concentration significantly reduced biomass in all populations. Oxidative stress biomarkers and enzyme activity levels indicated that populations from Salzgraben (Germany) and Inowrocław (Poland) demonstrated superior adaptation to varying salinity conditions. PCA revealed shoot length and biomass as key indicators of optimal growth, while H_2_O_2_ and MDA were the most useful for detecting subtle differences between populations under salt stress. NMDS showed that extreme salinity (1000 mM) caused the most significant divergence among populations, highlighting their distinct adaptation strategies.

### Growth performance under salt stress

Focusing on the salinity effect of morphometric parameters, we found that the shoot length (S-Length) of three populations (*Inow*, *Salz*, and *Soltq*) grown at 200 mM were the longest. A significant S-Length reduction was observed in *Ciech* population in 1000 mM NaCl, which was the shortest (on average by 37%, Table 1) within treatments (Table 1S). The root length (R-Length) of plants in *Inow* grown in 400 and 600 mM NaCl were the shortest within populations (Table 1). While the longest R-Length was found in *Soltq* population at 200 mM NaCl. Interestingly, the R-Length of all plants under salinity treatments was significantly longer than their corresponding S-Length (Table 1S).

**Table 1:** Effect of NaCl on the growth and water fraction of four inland populations of *Salicornia europaea*.

Trait	Population	NaCl concentration (mM)					
		0	200	400	600	800	1000
S-Length	Inowrocław	7.68^a^ ± 1.441	12.9^a^ ± 3.831	11.35^a^ ± 1.928	6.22^a^ ± 2.233	6.46^a^ ± 1.820	7.58^ab^ ± 1.973
	Ciechocinek	7.28^a^ ± 1.801	5.64^c^ ± 1.911	6.99^b^ ± 0.913	6.84^a^ ± 0.463	4.33^a^ ± 0.402	2.76^c^ ± 0.863
	Salzgraben	9.52^a^ ± 2.383	13.65^a^ ± 1.476	10.86^a^ ± 2.773	7.47^a^ ± 2.486	6.53^a^ ± 0.785	9.38^a^ ± 1.688
	Soltauquelle	7.82^a^ ± 2.407	16.02^a^ ± 2.134	10.86^a^ ± 2.062	6.83^a^ ± 2.117	4.33^a^ ± 1.859	5.34^bc^ ± 1.378
S-FW	Inowrocław	3.44^a^ ± 1.246	5.85^b^ ± 2.138	15.74^a^ ± 8.688	3.85^ab^ ± 2.255	2.78^b^ ± 0.897	2.18^a^ ± 0.403
	Ciechocinek	3.74^a^ ± 0.997	9.73^ab^ ± 6.346	10.10^ab^ ± 2.466	6.47^ab^ ± 1.055	1.09^b^ ± 0.286	0.50^b^ ± 0.270
	Salzgraben	7.26^a^ ± 2.154	15.15^a^ ± 9.710	15.82^a^ ± 8.160	12.11^a^ ± 4.071	10.98^a^ ± 4.438	4.41^a^ ± 1.322
	Soltauquelle	2.90^a^ ± 0.270	19.82^a^ ± 2.829	3.64^b^ ± 1.502	1.44^b^ ± 0.174	1.15^b^ ± 0.205	2.95^a^ ± 0.240
S-DW	Inowrocław	0.40^a^ ± 0.136	0.56^b^ ± 0.199	1.14^a^ ± 0.600	0.29^ab^ ± 0.193	0.20^ab^ ± 0.048	0.20^ab^ ± 0.041
	Ciechocinek	0.37^a^ ± 0.092	0.84^ab^ ± 0.667	0.62^ab^ ± 0.098	0.43^ab^ ± 0.050	0.09^b^ ± 0.032	0.05^c^ ± 0.026
	Salzgraben	0.61^a^ ± 0.188	0.96^ab^ ± 0.576	1.03^a^ ± 0.517	0.75^a^ ± 0.261	0.75^a^ ± 0.373	0.32^a^ ± 0.090
	Soltauquelle	0.32^a^ ± 0.075	1.18^a^ ± 0.154	0.35^b^ ± 0.103	0.10^b^ ± 0.006	0.08^b^ ± 0.016	0.22^bc^ ± 0.018
S-WF	Inowrocław	0.88^b^ ± 0.010	0.90^b^ ± 0.001	0.92^a^ ± 0.009	0.93^a^ ± 0.003	0.93^a^ ± 0.003	0.96^a^ ± 0.007
	Ciechocinek	0.90^ab^ ± 0.005	0.92^ab^ ± 0.010	0.94^a^ ± 0.005	0.93^a^ ± 0.003	0.92^a^ ± 0.020	0.92^b^ ± 0.012
	Salzgraben	0.92^a^ ± 0.001	0.93^a^ ± 0.019	0.93^a^ ± 0.000	0.94^a^ ± 0.004	0.93^a^ ± 0.010	0.93^b^ ± 0.006
	Solautquelle	0.89^b^ ± 0.030	0.94^a^ ± 0.001	0.90^b^ ± 0.020	0.93^a^ ± 0.008	0.93^a^ ± 0.003	0.93^b^ ± 0.001
R-Length	Inowrocław	18.50^a^ ± 4.390	15.78^b^ ± 2.398	16.83^b^ ± 1.331	17.68^b^ ± 3.217	20.92^a^ ± 5.211	19.15^bc^ ± 2.200
	Ciechocinek	21.74^a^ ± 5.097	22.27^b^ ± 3.970	25.91^a^ ± 4.410	25.15^a^ ± 1.631	20.81^a^ ± 2.892	15.96^c^ ± 4.295
	Salzgraben	17.79^a^ ± 2.511	17.69^b^ ± 4.543	26.57^a^ ± 3.292	23.81^ab^ ± 6.061	24.24^a^ ± 4.886	25.51^ab^ ± 4.413
	Soltauquelle	20.48^a^ ± 3.746	29.83^a^ ± 3.894	21.53^ab^ ± 3.332	23.77^ab^ ± 7.068	26.87^a^ ± 4.963	26.96^a^ ± 5.752
R-FW	Inowrocław	0.37^b^ ± 0.015	1.34^b^ ± 0.415	1.75^a^ ± 0.945	0.56^ab^ ± 0.284	0.58^ab^ ± 0.206	0.36^a^ ± 0.078
	Ciechocinek	0.43^b^ ± 0.067	1.09^b^ ± 0.497	0.89^b^ ± 0.275	0.40^ab^ ± 0.234	0.12^b^ ± 0.026	0.07^a^ ± 0.042
	Salzgraben	1.54^a^ ± 0.506	1.30^b^ ± 0.459	1.02^b^ ± 0.456	1.10^a^ ± 0.345	1.20^a^ ± 0.187	0.46^a^ ± 0.161
	Soltauquelle	0.56^b^ ± 0.141	2.21^a^ ± 0.467	0.49^b^ ± 0.123	0.21^b^ ± 0.029	0.23^b^ ± 0.058	0.44^a^ ± 0.114
R-DW	Inowrocław	0.07^b^ ± 0.011	0.15^a^ ± 0.051	0.20^a^ ± 0.124	0.05^a^ ± 0.010	0.05^ab^ ± 0.016	0.03^a^ ± 0.013
	Ciechocinek	0.06^b^ ± 0.026	0.12^a^ ± 0.025	0.08^b^ ± 0.021	0.04^a^ ± 0.023	0.01^b^ ± 0.006	0.03^a^ ± 0.010
	Salzgraben	0.16^a^ ± 0.051	0.13^a^ ± 0.050	0.11^b^ ± 0.050	0.10^a^ ± 0.029	0.12^a^ ± 0.031	0.05^a^ ± 0.014
	Soltauquelle	0.07^b^ ± 0.016	0.20^a^ ± 0.045	0.09^b^ ± 0.009	0.02^a^ ± 0.002	0.02^b^ ± 0.004	0.04^a^ ± 0.007
R-WF	Inowrocław	0.83^a^ ± 0.035	0.89^a^ ± 0.017	0.89^a^ ± 0.012	0.89^a^ ± 0.024	0.92^a^ ± 0.009	0.92^a^ ± 0.011
	Ciechocinek	0.87^a^ ± 0.037	0.87^a^ ± 0.028	0.90^a^ ± 0.019	0.89^a^ ± 0.005	0.89^a^ ± 0.032	0.53^b^ ± 0.240
	Salzgraben	0.89^a^ ± 0.008	0.91^a^ ± 0.007	0.89^a^ ± 0.005	0.91^a^ ± 0.003	0.90^a^ ± 0.011	0.89^a^ ± 0.008
	Soltauquelle	0.88^a^ ± 0.009	0.91^a^ ± 0.003	0.82^a^ ± 0.040	0.90^a^ ± 0.006	0.91^a^ ± 0.011	0.91^a^ ± 0.008

S = Shoots, R = roots, FW = Fresh weight, DW = dry weight, water fraction (WF). Average values with standard deviation (SD) are given length n = 5; FW, DW and WF n = 3. Two-way ANOVA with the Holm-Sidak method main effects between the four populations of *S. europaea* are marked by letters and are significantly different at *p*-value < 0.05.

The S-FW of the *Salz, Inow* and *Ciech* populations increased from 0 to 400 mM, but a significant decrease was found at 600, 800, and 1000 mM, as shown in Table 1S. At 200 mM NaCl the biomass in shoots and roots considerably increased (S-FW and R-FW, respectively) in *Soltq* compared to the other populations. Under severe salinity (1000 mM), *Salz, Soltq* and *Inow* demonstrated the highest biomass S-FW.

The R-FW at 1000 mM NaCl was the lowest observed across all populations. But, *Ciech* stands out with the shortest S-FW and R-FW. The water fraction in shoots (S-WF) increased along the salinity gradient in all the populations. *Ciech* exhibited the lowest S-WF and R-WF content (Table 1) (Table 1S).

### Oxidative stress biomarkers and osmotic stress defence

**Fig. 2:**
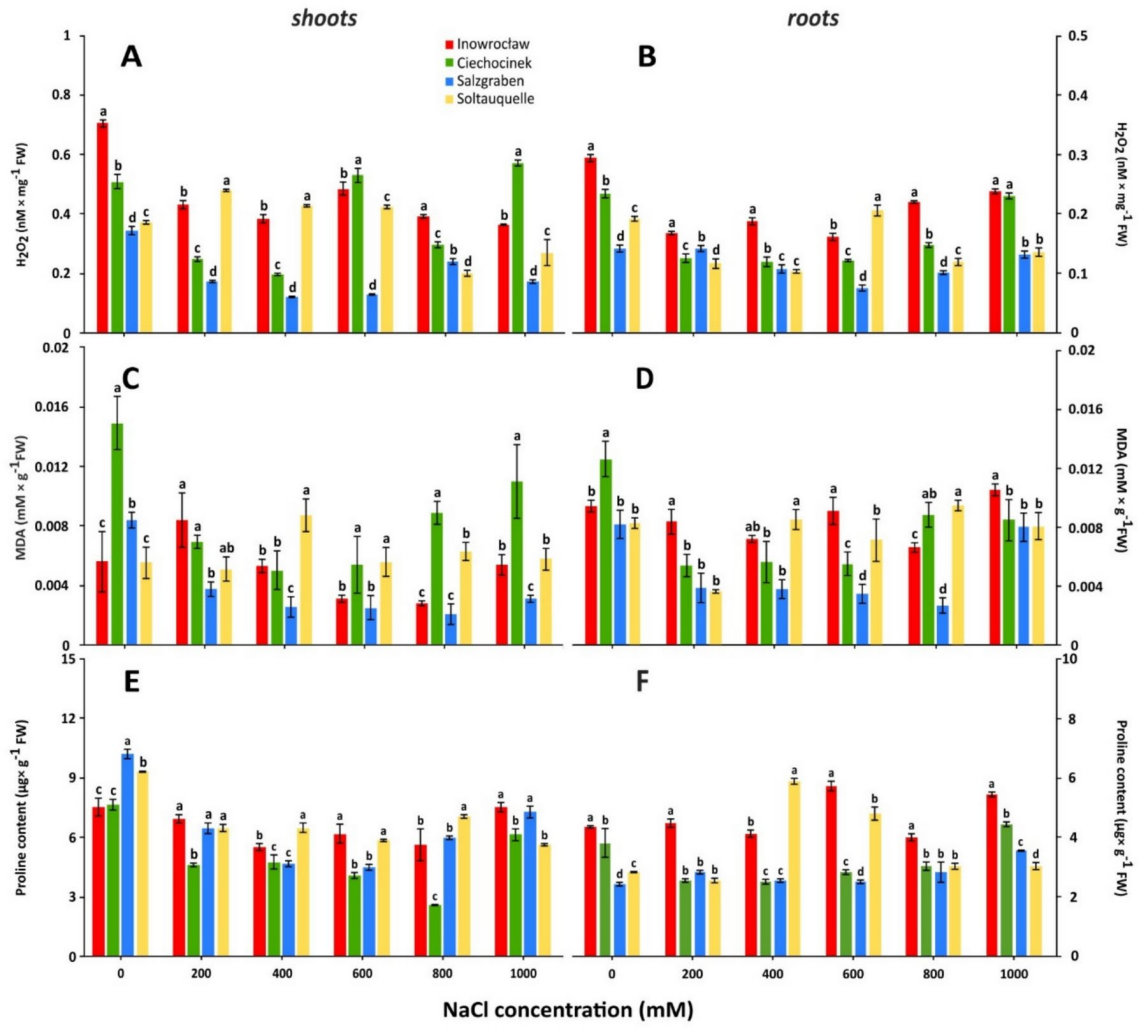
The hydrogen peroxide (H_2_O_2_) (**A**, **B**), malondialdehyde (MDA) (**C**, **D**) and proline (**E**, **F**) content in shoots and roots in *S. europaea* populations (Inowrocław, Ciechocinek, Salzgraben and Soltauquelle) under salt stress. Means and ± SD of replicates (n = 3). Different letters indicate significant differences (*p*-value < 0.05) between the four populations.

The two-way analysis of variance showed that population, salinity levels and their interaction (salinity x population) significantly differ in stress biomarkers of shoots and roots, with *p*-values < 0.001 (Table 1). Significant differences in S-H_2_O_2_ and R-H_*2*_O_*2*_ were detected within populations (Fig. 2A,B) and treatments (Table 2S). The lack of NaCl caused the highest S-H_2_O_2_ and R-H_2_O_2_ in *Inow* (Fig. 2) and (Table 2S). At 1000 mM NaCl, *Ciech* and *Salz* revealed the highest and lowest S-H_2_O_2_ content, respectively (Fig. 2A). Meanwhile, for R-H_2_O_2,_ the highest values were detected at 0 and 1000 mM in *Inow* and *Ciech*, respectively (Fig. 2B).

The malondialdehyde content in shoots and roots (S-MDA and R-MDA) was the highest in *Ciech* under no salinity treatments (Fig. 2C,D). Nevertheless, at severe salinity (1000 mM), *Ciech* and *Salz* exhibited the highest and lowest S-MDA content, respectively (Fig. 2C). At the root level, *Salz* had the lowest R-MDA from 400 to 800 mM NaCl (Fig. 2B,D). At the highest salinity level (1000 mM), *S. europaea* populations from *Inow* and *Salz* exhibited the highest S-PROL content, while *Ciech* and *Soltq* had the lowest levels (Fig. 2E). But in the roots, *Salz* showed the lowest proline content (R-PROL) at both 0 and 800 mM NaCl (Fig. 2F).

### Antioxidative stress defence

**Fig. 3:**
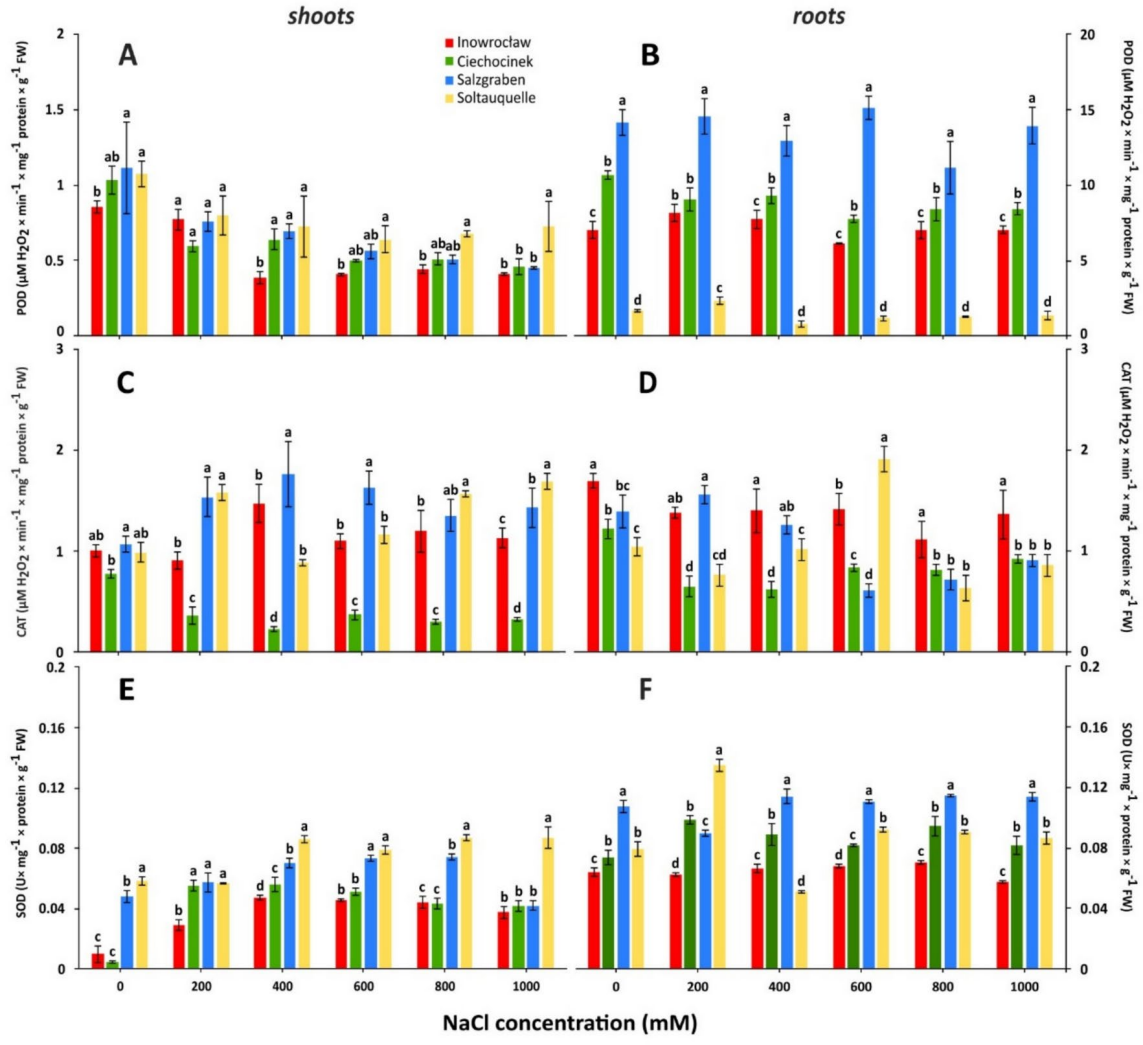
The activities of peroxidase guaiacol (POD) (**A**, **B**), catalase (CAT) (**C**, **D**) and superoxide dismutase (SOD) (**E**, **F**) in shoots and roots in *S. europaea* populations (Inowrocław, Ciechocinek, Salzgraben and Soltauquelle) under salt stress. Means and ± SD of replicates (n = 3). Different letters indicate significant differences (*p*-value < 0.05) between the four populations.

The two-way analysis of variance showed that population, salinity levels and their interaction (salinity × population) significantly differ in antioxidative enzyme activity of shoots and roots, with *p*-values < 0.001 (Table 1). Peroxidase activity in shoots (S-POD and R-POD) differs from non-salinity to higher salinity treatments within populations and within salinity treatments, (Table 1 and 2S). The significant highest POD activity within populations occurred in the S-POD of the *Soltq* population at 1000 mM through the salinity treatments. In turn, the significantly lowest activity of this enzyme was observed in the S-POD of *Inow* (0 and 400 mM) (Fig. 3A). The analysis of enzyme activity in the roots (R-POD) revealed the lowest and highest levels in *Soltq* and *Salz* respectively (Fig. 3B). Within Poland populations, *Inow* exhibited less R-POD than *Ciech*.

The lowest catalase activity in shoots (S-CAT) was found in *Ciech* for all salinity treatments (Fig. 3C; Table 2S), while the highest S-CAT activities were found in *Salz* (200,400, 600 and 800 mM NaCl) and *Soltq* (200, 800 and 1000 mM of NaCl). Among the tested enzyme activities, it is worth to highlight that this enzyme showed peak activity in *Inow*, *Salz* and *Soltq* populations (Fig. 3C). For instance, the highest R-CAT was noted in the *Inow* plants for almost all the treatments. While the lowest R-CAT was found in *Ciech* at 200 and 400 mM NaCl (Fig. 3D).

Regarding SOD in shoots (S-SOD), plants from the *Ciech* and *Inow* populations grown without NaCl had the lowest activity within treatments and compared to German populations (Fig. 3E; Table 2S). At higher salinities *Soltq* exhibited the highest activity of S-SOD and R-SOD while *Inow* had the lowest activity (Fig. 3F).

### Differences in stress adaptations between four populations

The two-way analysis of variance showed that populations, salinity levels and their interaction (salinity × population) significantly differ in antioxidative enzymes activity of shoots and roots, with *p*-values < 0.001 (Table 2).

**Table 2:** Two-way ANOVA mean squares for morphological parameters, salinity stress biomarkers and activity of antioxidant enzymes in shoots and roots of the four inland populations of *S. europaea* and six salt concentrations.

	*df*	Mean squares									
		S-Length	S-FW	S-DW	S-WF	S-POD	S-CAT	S-SOD	S-H_2_O_2_	S-MDA	S-PROL
Salinity level	5	125.94**	209.86**	0.93**	0.002**	0.456**	0.047*	0.002**	0.068**	2.8 × 10^–5^**	21.31**
Population	3	87.61**	138.67**	0.496**	0.0006*	0.164**	4.06**	0.006**	0.224**	7.9 × 10^–5^**	12.59**
Salinity level × population	15	17.63**	44.65**	0.143*	0.0006**	0.018^n.s^	0.235**	0.0003**	0.038**	1.6 × 10^–5^**	2.87**
Error	48	12.79	14.59	0.076	0.0002	0.010	0.016	1.25 × 10^–5^	0.0002	1.3 × 10^–6^	0.079

	*df*	Mean squares									
		R-Length	R-FW	R-DW	R-WF	R-POD	R-CAT	R-SOD	R-H_2_O_2_	R-MDA	R-PROL
Salinity level	5	236.22**	2.09**	0.024**	0.015**	3.92**	0.367**	0.0005**	0.013**	0.00003**	2.11**
Population	3	33.20^n.s^	1.15**	0.009**	0.021**	459.40**	0.941**	0.0057**	0.029**	4.25 × 10^–5^**	12.15**
Salinity level × population	15	62.20**	0.51**	0.004**	0.019**	2.48**	0.316**	0.0008**	0.004**	9.69 × 10^–6^**	2.38**
Error	48	17.74	0.113	0.001	0.003	0.483	0.014	1.29 × 10^–5^	3.26 × 10^–5^	7.03 × 10^–7^	0.025

S = Shoots, R = roots, FW = fresh weight, DW = dry weight, water fraction (WF), enzyme activity: peroxidase (POD), catalase (CAT), superoxide dismutase (SOD), salt-stress biomarkers: hydrogen peroxide (H_2_O_2_) malondialdehyde (MDA) and proline (PROL).

n.s.: non-significant; *: significant at *p* < 0.05; **: significant at *p* < 0.001.

**Fig. 4:**
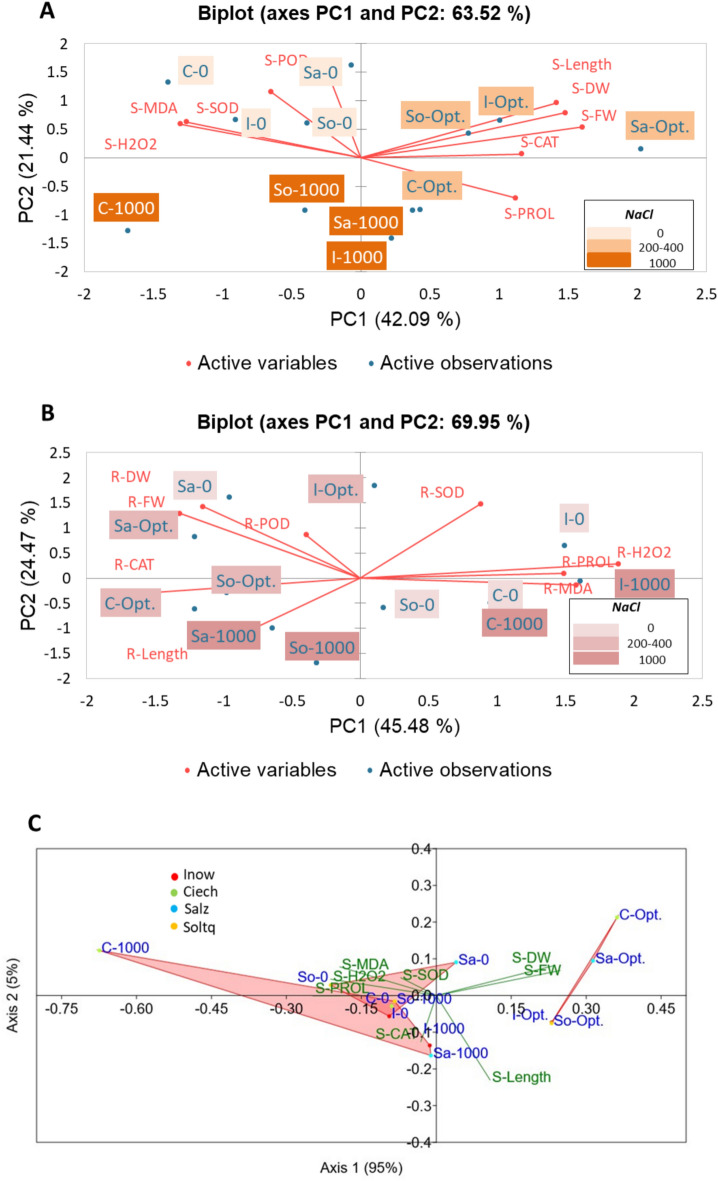
Principal component analysis (PCA) of the four populations under non-salinity (0 mM), optimum (Opt: 200–400 mM) and severe salinity (1000 mM) with morphometry and biochemical variables tested in shoots (**A**) and roots (**B**). Non-metric multidimensional scaling ordination diagram (NMDS with Bray–Curtis dissimilarity) of the four populations. Axis 1 (95%) accounts for variance in the measured parameters, Axis 2 (5%) represents differences in population responses under non-salinity (0 mM), optimum (Opt: 200–400 mM) and severe salinity (1000 mM) (**C**). Abbreviations: S (shoots), fresh weight (S-FW), dry weight (S-DW), enzyme activity: peroxidase (S-POD), catalase (S-CAT), superoxide dismutase (S-POD), salt-stress biomarkers: hydrogen peroxide (S-H_2_O_2_) malondialdehyde (S-MDA) and proline (S-PROL). I: Inowrocław, C: Ciechocinek, Sa: Salzgraben and So: Soltauquelle.

The first PCA (Fig. 4A) related to shoot parameters showed that the two PCs explained over 63.5% of the total variation which is meaningful according to the dataset complexity of our study. It is showing that at shoot level, *Ciech* population was the most affected under severe salinity conditions. Meanwhile, *Salz* stands out as the population with the best development under optimum conditions. The salinity levels were well separated on PC1, which accounted for more than 42% of the total variation. We observed that the non-salinity and highest-salinity treatments, considered as stress conditions, were positioned on the negative side of PC1. At the same time, the optimum treatment conditions fell on the upper positive side. In this study, S-H_2_O_2_ and S-MDA were the most suitable to detect subtle differences between the four populations when subjected to salt stress conditions, confirming each one as a distinct ecotype. The second PCA (Fig. 4B) for root parameters, explained over 69.95%, it shows that at the root level, German populations have a better salt mechanism tolerance than Polish populations under extreme salinity conditions, whether lack of salinity or severe salinity *Salz* and *Solt* expressed higher root length and lower stress biomarkers content. *Inow* and *Ciech* experienced higher content of stress biomarkers for both, no salinity and severe salinity.

To picture the distance metric value of each population in relation to their distances through the salinity treatments, non-metric multidimensional scaling (NMDS) was performed (Fig. 4C). In the NMDS, the first ordination axis relates to the stress gradient. Samples are distributed from the stress condition treatments on the left (0 mM and 1000 mM NaCl) to the right side of the diagram, where the optimal conditions (200–400 mM NaCl) are located. The first axis accounts for 95% of the variance in the measured parameters. The second NMDS axis relates to differences in population responses and only accounts for 5% of the total variance. The shortest separation between treatments was found at non-salinity and optimum salinity, while the most significant separation was noted in severe salinity (1000 mM NaCL). *Ciech* population stands out as the furthest from the severe salinity group, while *Salz*, *Inow* and *Soltq* demonstrated a better salt tolerance (Fig. 4C).

## Discussion

*Salicornia europaea* employs adaptative cellular mechanisms to thrive in saline environments. Understanding how environmental factors influence salt-induced biochemical responses is crucial for deciphering adaptation strategies. Analysing biochemical dynamics across populations helps identify early salinity stress biomarkers, adaptation mechanisms and provides new insight about the restoration strategy of saline areas. This study examined biomass and biochemical responses in four *S. europaea* populations under varying NaCl conditions.

### Adaptive mechanisms and growth performance

Salinity significantly influenced biomass production, with each population exhibiting distinct salt tolerance strategies. Analysis of variance revealed that salinity levels, population differences, and their interactions significantly affected shoot and root parameters (Table 1). Optimal growth occurred at 200–400 mM NaCl^18,19^. Under severe salinity, *Salz, Inow*, and *Soltq* performed best, suggesting effective salt adaptation mechanisms, including organic solute accumulation and ion regulation to maintain turgor pressure^21,43^.

*Salz* and *Soltq* populations demonstrated superior root growth (R-Length and R-FW) under extreme salinity (1000 mM), while *Inow* and *Ciech* showed weaker root-level adaptations. Morphological plasticity in root systems is crucial for nutrient uptake and salinity tolerance^44,45^. Root adjustments help prevent salt accumulation and function as environmental sensors during salt stress adaptation. *S. europaea* exhibits exceptional salt resistance^11,15^, capable of seawater irrigation (0.5 M NaCl) for optimal growth, though excessive concentrations slow development due to salt-induced stress^22^. Low and high salinity levels trigger population-specific stress responses^33,46^.

### Biochemical stress biomarkers and antioxidant responses

Variations in hydrogen peroxide (H_2_O_2_) levels in shoots and roots reflected population-specific stress responses. H_2_O_2_ acts as a signaling molecule for stress adaptation. Recent studies recognized that reactive oxygen species (ROS), such as H_2_O_2_, are essential regulators of plant growth and development, particularly in root system architecture and adaptation to environmental challenges^28–30^. But, at excessive levels they can cause oxidative damage^28,47^. The *Inow* population showed the highest S-H_2_O_2_ at 0 mM, while *Ciech* peaked at 1000 mM, indicating stress sensitivity. The *Salz* population exhibited the lowest S-H_2_O_2_ levels across all treatments, suggesting superior salt adaptation. The present findings align with Su et al.^28^, who stated that ROS regulation, particularly H_2_O_2_, plays a critical role in root system architecture and environmental adaptation.

Malondialdehyde (MDA) levels, an indicator of lipid peroxidation and oxidative stress^48^, varied among populations. *Salz* displayed the lowest MDA levels, while *Ciech* had the highest. Reduced MDA suggests enhanced oxidative damage protection, aligning with findings in other other taxa of *Salicornia*^49^. Proline, an important osmoprotectant, showed significant variations across populations, with *Inow* accumulating the highest levels at severe salinity, likely aiding water retention and plant cells stress mitigation^21,50^.

### Antioxidant enzymes and population-specific adaptations

Antioxidant enzyme activity varied significantly, with *Salz* and *Soltq* populations exhibiting the most effective enzymatic defenses. Peroxidase (POD) activity was the highest in *Soltq* shoots and *Salz* roots, correlating with their superior performance under saline conditions^51^. Catalase (CAT) and superoxide dismutase (SOD) activity also fluctuated, with *Inow* demonstrating elevated root CAT at 1000 mM, indicative of its specific salt tolerance mechanism^48^. However, Polish populations (*Inow, Ciech*) showed lower antioxidant enzyme activity, making them more vulnerable to oxidative stress.

Recent studies^52^ have questioned the reliability of enzymatic antioxidants as definitive stress tolerance markers in halophytes. Therefore, future research should adopt a more comprehensive approach, integrating total antioxidant capacity assessments, including non-enzymatic antioxidants like phenolics and flavonoids, alongside ROS-scavenging efficiency.

### Principal component analysis and population variability

PCA and NMDS analyses confirmed population-specific salt adaptation strategies. *Salz Soltq* and *Inow* populations consistently exhibited superior tolerance, while *Ciech* showed the highest stress sensitivity under severe salinity. Population responses are influenced by genetic and environmental factors, demanding further research on salt adaptation mechanisms.

### Effect of salinity on nutritional content

Salinity significantly impacts the nutritional composition of *S. europaea*^4,15,53^, influencing its biochemical responses and adaptation strategies. Elevated salinity levels have been shown to enhance the accumulation of beneficial secondary metabolites, such as phenolic compounds and flavonoids, which contribute to the plant’s antioxidant capacity and stress tolerance^54^. For instance, studies have identified high levels of flavonoids and their glycosides, including quercetin and kaempferol derivatives in *S. europaea* under moderate and higher saline conditions^10^. The ability to synthesize these compounds varies among populations and is closely linked to their salt adaptation mechanisms. Therefore, populations such as *Salz, Inow*, and *Soltq*, which exhibit better adaptation moderate to higher saline environments, can offer higher nutritional value. Increased salinity also influences the mineral content of *S. europaea*, affecting its overall nutritional value. The plant is known to accumulate significant amounts of beneficial minerals for human intake, such as sodium (Na), potassium (K), calcium (Ca), and magnesium (Mg), iodine (I), vitamin B3 and C along with antioxidant compounds, when exposed to saline environments^15,53,54^. Understanding these changes provides insights into potential applications of *S. europaea* in saline agriculture and functional food production.

### Future research directions

Distinct adaptation strategies among populations underscore the need for further studies integrating genetic analysis, environmental influences, and non-enzymatic antioxidant assessments. Plants from the *Salz, Soltq*, and *Inow* populations demonstrated superior salt adaptation, confirming their resilience to high salinity. Future research should explore maternal origin influences and epigenetic factors shaping stress responses, ensuring a comprehensive understanding of *S. europaea* adaptation mechanisms.

## Conclusions

Our study reveals distinct salt stress responses among the four inland populations of *S. europaea*, showing that while each population possesses unique adaptive mechanisms, *Salz* and *Inow* exhibit the highest resilience under severe salinity. These populations may be particularly well suited for saline land restoration and offer greater nutritional value. In contrast, *Ciech* is the most vulnerable, potentially serving as an indicator of an ecologically impacted maternal area. These findings emphasize the significant role of environmental and maternal origin factors in shaping the salt tolerance of each ecotype.

Beyond stress adaptation, salinity may also influence the nutritional composition of *S. europaea*, enhancing its accumulation of beneficial secondary metabolites, such as phenolics and flavonoids, which contribute to its antioxidant properties. Additionally, high salinity increases essential mineral content, enhancing its potential as a functional food and as a candidate for saline agriculture.

Understanding the relationship between genetic origins, environmental influences, and individual responses to salt stress offers valuable insights into the species’ ecological selection and agricultural applications. Further research, particularly on epigenetic variability, will be crucial for uncovering the mechanisms governing salt stress adaptation and optimizing *S. europaea* for land reclamation, functional food production, and sustainable saline agriculture.

## Supplementary Information

Supplementary Information.

## Data Availability

Data is provided within the manuscript or supplementary information files.
