# Supplementary material for: The local environment influences salt tolerance differently in four Salicornia europaea L. inland populations
Source: Sci Rep. 2025 Apr 16;15:13128. doi: 10.1038/s41598-025-97394-5 (PMC12003738; doi:10.1038/s41598-025-97394-5)
Supplement: Supplementary file 1 — Supplementary Information. [file 41598_2025_97394_MOESM1_ESM.docx]

Table 1S. Effect of tested NaCl concentrations on the growth and water fraction of four inland populations of *Salicornia europaea*.

|  | |  | | | NaCl concentration (mM) | | | | | | | | | |  |
| --- | --- | --- | --- | --- | --- | --- | --- | --- | --- | --- | --- | --- | --- | --- | --- |
| Trait | **Population** | | | **0** | | | **200** | **400** | | **600** | **800** | | **1000** | |  |
| S-Length | Inowrocław  Ciechocinek  Saltzgraben  Soltquelle | | 7.68^bc^ ± 1.441  7.28^a^ ± 1.801  9.52^bc^ ± 2.383  7.82^bc^ ± 2.407 | | | 12.9^a^ ± 3.831  5.64^ab^ ± 1.911  13.65^a^ ± 1.476  16.02^a^ ± 2.134 | | 11.35^ab^ ± 1.928  6.99^a^ ± 0.913  10.86^ab^ ± 2.773  10.86^b^ ± 2.062 | 6.22^c^ ± 2.233  6.84^a^ ± 0.463  7.47^bc^ ± 2.486  6.83^bc^ ± 2.117 | | | 6.46^bc^ ±1.820  4.33^ab^ ± 0.402  6.53^c^ ± 0.785  4.33^c^ ± 1.859 | | 7.58^bc^ ± 1.973  2.76^b^ ± 0.863  9.38^bc^ ± 1.688  5.34^c^ ± 1.378 | |
| S-FW | Inowrocław  Ciechocinek  Saltzgraben  Soltquelle | | 3.44^b^ ± 1.246  3.74^ab^ ± 0.997  7.26^ab^ ± 2.154  2.90^b^ ± 0.270 | | | 5.85^b^ ± 2.138  9.73^a^ ± 6.346  15.15^a^ ± 9.710  19.82^a^ ± 2.829 | | 15.74^a^ ± 8.688  10.10^a^ ± 2.466  15.82^a^ ± 8.160  3.64^b^ ± 1.502 | 3.85^b^ ± 2.255  6.47^ab^ ± 1.055  12.11^ab^ ± 4.071  1.44^b^ ± 0.174 | | | 2.78^b^ ± 0.897  1.09^ab^ ± 0.286  10.98^ab^ ± 4.438  1.15^b^ ± 0.205 | | 2.18^b^ ± 0.403  0.50^b^ ± 0.270  4.41^b^ ± 1.322  2.95^b^ ± 0.240 | |
| S-DW | Inowrocław  Ciechocinek  Saltzgraben  Soltquelle | | 0.40^b^ ± 0.136  0.37^ab^ ± 0.092  0.61^ab^ ± 0.188  0.32^ab^ ± 0.075 | | | 0.56^ab^ ± 0.199  0.84^a^ ± 0.667  0.96^ab^ ± 0.576  1.18^a^ ± 0.154 | | 1.14^a^ ± 0.600  0.62^ab^ ± 0.098  1.03^a^ ± 0.517  0.35^ab^ ± 0.103 | 0.29^b^ ± 0.193  0.43^ab^ ± 0.050  0.75^ab^ ± 0.261  0.10^b^ ± 0.006 | | | 0.20^b^ ± 0.048  0.09^b^ ± 0.032  0.75^ab^ ± 0.373  0.08^b^ ± 0.016 | | 0.20^b^ ± 0.041  0.05^b^ ± 0.026  0.32^b^ ± 0.090  0.22^ab^ ± 0.018 | |
| S-WF | Inowrocław  Ciechocinek  Saltzgraben  Soltquelle | | 0.88^c^ ± 0.010  0.90^b^ ± 0.005  0.92^a^ ± 0.001  0.89^c^ ± 0.030 | | | 0.90^bc^ ± 0.001  0.92^ab^ ± 0.010  0.93^a^ ± 0.019  0.94^a^ ± 0.001 | | 0.92^ab^ ± 0.009  0.94^a^ ± 0.005  0.93^a^ ± 0.000  0.90^bc^ ± 0.020 | 0.93^ab^ ± 0.003  0.93^a^ ± 0.003  0.94^a^ ± 0.004  0.93^ab^ ± 0.008 | | | 0.93^ab^ ± 0.003  0.92^ab^ ± 0.020  0.93^a^ ± 0.010  0.93^ab^ ± 0.003 | | 0.96^a^ ± 0.007  0.92^ab^ ± 0.012  0.93^a^ ± 0.006  0.93^ab^ ± 0.001 | |
| R-Length | Inowrocław  Ciechocinek  Saltzgraben  Soltquelle | | 18.50^a^ ± 4.390  21.74^ab^ ± 5.097  17.79^b^ ± 2.511  20.48^b^ ± 3.746 | | | 15.78^a^ ± 2.398  22.27^ab^ ± 3.970  17.69^b^ ± 4.543  29.83^a^ ± 3.894 | | 16.83^a^ ± 1.331  25.91^a^ ± 4.410  26.57^a^ ± 3.292  21.53^b^ ± 3.332 | 17.68^a^ ± 3.217  25.15^a^ ± 1.631  23.81^ab^ ± 6.061  23.77^ab^ ± 7.068 | | | 20.92^a^ ± 5.211  20.81^ab^ ± 2.892  24.24^ab^ ± 4.886  26.87^ab^ ± 4.963 | | 19.15^a^ ± 2.200  15.96^b^ ± 4.295  25.51^ab^ ± 4.413  26.96^ab^ ± 5.752 | |
| R-FW | Inowrocław  Ciechocinek  Saltzgraben  Soltquelle | | 0.37^cd^ ± 0.015  0.43^ab^ ± 0.067  1.54^a^ ± 0.506  0.56^b^ ± 0.141 | | | 1.34^abc^ ± 0.415  1.09^a^ ± 0.497  1.30^a^ ± 0.459  2.21^a^ ± 0.467 | | 1.75^a^ ± 0.945  0.89^ab^ ± 0.275  1.02^ab^ ± 0.456  0.49^b^ ± 0.123 | 0.56^bcd^ ± 0.284  0.40^ab^ ± 0.234  1.10^ab^ ± 0.345  0.21^b^ ± 0.029 | | | 0.58^cd^ ± 0.206  0.12^b^ ± 0.026  1.20^ab^ ± 0.187  0.23^b^ ± 0.058 | | 0.36^d^ ± 0.078  0.07^b^ ± 0.042  0.46^b^ ± 0.161  0.44^b^ ± 0.114 | |
| R-DW | Inowrocław  Ciechocinek  Saltzgraben  Soltquelle | | 0.07^b^ ± 0.011  0.06^ab^ ± 0.026  0.16^a^ ± 0.051  0.07^b^ ± 0.016 | | | 0.15^ab^ ± 0.051  0.12^a^ ± 0.025  0.13^ab^ ± 0.050  0.20^a^ ± 0.045 | | 0.20^a^ ± 0.124  0.08^ab^ ± 0.021  0.11^ab^ ± 0.050  0.09^b^ ± 0.009 | 0.05^b^ ± 0.010  0.04^ab^ ± 0.023  0.10^ab^ ± 0.029  0.02^b^ ± 0.002 | | | 0.05^b^ ± 0.016  0.01^b^ ± 0.006  0.12^ab^ ± 0.031  0.02^b^ ± 0.004 | | 0.03^b^ ± 0.013  0.03^b^ ± 0.010  0.05^b^ ± 0.014  0.04^b^ ± 0.007 | |
| R-WF | Inowrocław  Ciechocinek  Saltzgraben  Soltquelle | | 0.83^a^ ± 0.035  0.87^a^ ± 0.037  0.89^a^ ± 0.008  0.88^a^ ± 0.009 | | | 0.89^a^ ± 0.017  0.87^a^ ± 0.028  0.91^a^ ± 0.007  0.91^a^ ± 0.003 | | 0.89^a^ ± 0.012  0.90^a^ ± 0.019  0.89^a^ ± 0.005  0.82^a^ ± 0.040 | 0.89^a^ ± 0.024  0.89^a^ ± 0.005  0.91^a^ ± 0.003  0.90^a^ ± 0.006 | | | 0.92^a^ ± 0.009  0.89^a^ ± 0.032  0.90^a^ ± 0.011  0.91^a^ ± 0.011 | | 0.92^a^ ± 0.011  0.53^b^ ± 0.240  0.89^a^ ± 0.008  0.91^a^ ± 0.008 | |

Abbreviations: S and R stand for shoots and roots, respectively: FW - fresh weight, DW - dry weight, WF - water fraction. Average values with standard deviation (SD) are given: length n=5; FW, DW and WF n=3. Two-way ANOVA with the Holm-Sidak method main effects between the tested salt concentrations in each population of *Salicornia europaea* are marked by letters per row and are significantly different at p-value < 0.05.

Table 2S. The activity of antioxidant enzymes and the content of salinity stress biomarkers in shoots and roots of four inland populations of *Salicornia europaea*.

|  |  | NaCl concentration (mM) | | | | | | | | | |
| --- | --- | --- | --- | --- | --- | --- | --- | --- | --- | --- | --- |
| Trait | **Population** | **0** | **200** | | **400** | **600** | | | **800** | **1000** | |
| S-POD | Inowrocław  Ciechocinek  Saltzgraben  Soltquelle | 0.85^a^ ± 0.041  1.03^a^ ± 0.092  1.11^a^ ± 0.304  1.07^a^ ± 0.083 | | 0.77^a^ ± 0.072  0.60^b^ ± 0.034  0.76^b^ ± 0.065  0.80^b^ ± 0.130 | 0.39^b^ ± 0.041  0.64^b^ ± 0.066  0.69^b^ ± 0.049  0.72^b^ ± 0.202 | | 0.41^b^ ± 0.009  0.50^b^ ± 0.010  0.56^b^ ± 0.046  0.64^b^ ± 0.092 | 0.44^b^ ± 0.028  0.51^b^ ± 0.042  0.51^b^ ± 0.026  0.68^b^ ± 0.019 | | | 0.41^b^ ± 0.009  0.46^b^ ± 0.055  0.45^b^ ± 0.007  0.72^b^ ± 0.167 |
| S-CAT | Inowrocław  Ciechocinek  Saltzgraben  Soltquelle | 1.00^b^ ± 0.064  0.77^a^ ± 0.043  1.07^c^ ± 0.079  0.99^bc^ ± 0.096 | | 0.91^b^ ± 0.087  0.36^b^ ± 0.090  1.53^ab^ ± 0.196  1.59^a^ ± 0.080 | 1.47^a^ ± 0.192  0.23^b^ ± 0.024  1.76^a^ ± 0.325  0.88^c^ ± 0.029 | | 1.10^b^ ± 0.075  0.37^b^ ± 0.050  1.63^ab^ ± 0.165  1.16^bc^ ± 0.084 | 1.20^ab^ ± 0.204  0.30^b^ ± 0.021  1.35^bc^ ± 0.157  1.57^a^ ± 0.027 | | | 1.12^b^ ± 0.094  0.33^b^ ± 0.022  1.43^b^ ± 0.199  1.69^a^ ± 0.079 |
| S-SOD | Inowrocław  Ciechocinek  Saltzgraben  Soltquelle | 0.01^e^ ± 0.005  0.004^d^ ± 0.001  0.05^c^ ± 0.004  0.06^d^ ± 0.003 | | 0.03^d^ ± 0.004  0.06^ab^ ± 0.004  0.06^b^ ± 0.006  0.06^d^ ± 0.0003 | 0.05^a^ ± 0.002  0.06^a^ ± 0.005  0.07^a^ ± 0.003  0.09^abc^ ± 0.002 | | 0.05^ab^ ± 0.001  0.05^ab^ ± 0.002  0.07^a^ ± 0.002  0.08^c^ ± 0.003 | 0.04^abc^ ± 0.004  0.04^c^ ± 0.004  0.07^a^ ± 0.002  0.09^ab^ ± 0.002 | | | 0.04^c^ ± 0.004  0.04^c^ ± 0.004  0.04^c^ ± 0.003  0.09^a^ ± 0.007 |
| S-H_2_O_2_ | Inowrocław  Ciechocinek  Saltzgraben  Soltquelle | 0.70^a^ ± 0.013  0.51^b^ ± 0.024  0.34^a^ ± 0.014  0.37^c^ ± 0.005 | | 0.43^c^ ± 0.014  0.25^d^ ± 0.010  0.17^c^ ± 0.005  0.48^a^ ± 0.003 | 0.38^d^ ± 0.012  0.20^e^ ± 0.005  0.12^d^ ± 0.002  0.43^b^ ± 0.005 | | 0.48^b^ ± 0.024  0.53^b^ ± 0.024  0.13^d^ ± 0.003  0.42^b^ ± 0.006 | 0.39^d^ ± 0.006  0.30^c^ ± 0.008  0.24^b^ ± 0.009  0.20^e^ ± 0.010 | | | 0.36^d^ ± 0.002  0.57^a^ ± 0.009  0.17^c^ ± 0.006  0.27^d^ ± 0.043 |
| S-MDA | Inowrocław  Ciechocinek  Saltzgraben  Soltquelle | 0.006^b^ ± 0.0020  0.015^a^ ± 0.0018  0.008^a^ ± 0.0005  0.006^b^ ± 0.0010 | | 0.008^a^ ± 0.0018  0.007^c^ ± 0.0005  0.004^b^ ± 0.0005  0.005^b^ ± 0.0008 | 0.005^bc^ ± 0.0005  0.005^c^ ± 0.0013  0.003^b^ ± 0.0007  0.009^a^ ± 0.0011 | | 0.003^bc^ ± 0.0002  0.005^bc^ ± 0.0019  0.002^b^ ± 0.0008  0.006^b^ ± 0.0010 | 0.003^c^ ± 0.0002  0.009^b^ ± 0.0008  0.002^b^ ± 0.0007  0.006^ab^ ± 0.0006 | | | 0.005^bc^ ± 0.0007  0.011^b^ ± 0.0025  0.003^b^ ± 0.0002  0.006^b^ ± 0.0007 |
| S-PROL | Inowrocław  Ciechocinek  Saltzgraben  Soltquelle | 7.52^a^ ± 0.423  7.64^a^ ± 0.261  10.18^a^ ± 0.222  9.31^a^ ± 0.040 | | 6.93^a^ ± 0.190  4.59^cd^ ± 0.087  6.47^c^ ± 0.259  6.47^bc^ ± 0.176 | 5.48^c^ ± 0.180  4.75^c^ ± 0.346  4.67^d^ ± 0.155  6.48^bc^ ± 0.235 | | 6.19^b^ ± 0.494  4.09^d^ ± 0.151  4.49^d^ ± 0.171  5.88^cd^ ± 0.080 | 5.63^bc^ ± 0.827  2.59^e^ ± 0.050  5.97^c^ ± 0.107  7.03^b^ ± 0.085 | | | 7.51^a^ ± 0.221  6.15^b^ ± 0.290  7.32^b^ ± 0.280  5.64^d^ ± 0.055 |
| R-POD | Inowrocław  Ciechocinek  Saltzgraben  Soltquelle | 7.00^ab^ ± 0.547  10.70^a^ ± 0.289  14.19^ab^ ± 0.850  1.66^a^ ± 0.064 | | 8.14^a^ ± 0.582  9.04^ab^ ± 0.775  14.55^ab^ ± 1.186  2.34^a^ ± 0.245 | 7.76^ab^ ± 0.610  9.26^ab^ ± 0.525  12.95^b^ ± 1.008  0.81^a^ ± 0.206 | | 6.10^b^ ± 0.037  7.78^b^ ± 0.231  15.15^a^ ± 0.767  1.17^a^ ± 0.171 | 7.01^ab^ ± 0.571  8.40^b^ ± 0.797  11.19^c^ ± 1.729  1.27^a^ ± 0.024 | | | 7.06^ab^ ± 0.235  8.44^b^ ± 0.413  13.94^ab^ ± 1.200  1.36^a^ ± 0.288 |
| R-CAT | Inowrocław  Ciechocinek  Saltzgraben  Soltquelle | 1.69^a^ ± 0.07  1.21^a^ ± 0.10  1.39^ab^ ± 0.16  1.04^b^ ± 0.09 | | 1.38^bc^ ± 0.06  0.64^bc^ ± 0.10  1.56^a^ ± 0.09  0.76^c^ ± 0.11 | 1.40^b^ ± 0.22  0.62^c^ ± 0.08  1.26^b^ ± 0.09  1.01^bc^ ± 0.11 | | 1.41^b^ ± 0.16  0.83^bc^ ± 0.03  0.61^d^ ± 0.06  1.91^a^ ± 0.13 | 1.11^c^ ± 0.18  0.81^bc^ ± 0.05  0.71^cd^ ± 0.10  0.62^c^ ± 0.13 | | | 1.36^bc^ ± 0.24  0.92^b^ ± 0.04  0.90^c^ ± 0.06  0.85^bc^ ± 0.11 |
| R-SOD | Inowrocław  Ciechocinek  Saltzgraben  Soltquelle | 0.06^ab^ ± 0.003  0.07^d^ ± 0.005  0.11^a^ ± 0.004  0.08^c^ ± 0.005 | | 0.06^ab^ ± 0.001  0.10^a^ ± 0.003  0.09^b^ ± 0.002  0.13^a^ ± 0.004 | 0.07^ab^ ± 0.003  0.09^bc^ ± 0.007  0.11^a^ ± 0.005  0.05^d^ ± 0.001 | | 0.07^a^ ± 0.001  0.08^cd^ ± 0.001  0.11^a^ ± 0.001  0.09^b^ ± 0.002 | 0.07^a^ ± 0.001  0.09^ab^ ± 0.007  0.11^a^ ± 0.001  0.09^b^ ± 0.001 | | | 0.06^b^ ± 0.001  0.08^c^ ± 0.006  0.11^a^ ± 0.003  0.09^b^ ± 0.004 |
| R-H_2_O_2_ | Inowrocław  Ciechocinek  Saltzgraben  Soltquelle | 0.29^a^ ± 0.006  0.23^a^ ± 0.007  0.14^a^ ± 0.006  0.19^b^ ± 0.004 | | 0.17^ef^ ± 0.002  0.13^de^ ± 0.008  0.14^ab^ ± 0.006  0.12^de^ ± 0.003 | 0.19^d^ ± 0.006  0.12^e^ ± 0.008  0.11^c^ ± 0.007  0.10^e^ ± 0.003 | | 0.16^f^ ± 0.006  0.12^de^ ± 0.002  0.08^d^ ± 0.005  0.21^a^ ± 0.009 | 0.22^c^ ± 0.003  0.15^c^ ± 0.004  0.10^c^ ± 0.003  0.12^d^ ± 0.006 | | | 0.24^b^ ± 0.004  0.23^ab^ ± 0.005  0.13^ab^ ± 0.006  0.14^c^ ± 0.007 |
| R-MDA | Inowrocław  Ciechocinek  Saltzgraben  Soltquelle | 0.009^a^ ± 0.0004  0.013^a^ ± 0.0012  0.008^a^ ± 0.0010  0.008^ab^ ± 0.0004 | | 0.009^ab^ ± 0.0008  0.005^c^ ± 0.0007  0.004^c^ ± 0.0010  0.004^c^ ± 0.0002 | 0.007^bc^ ± 0.0002  0.006^c^ ± 0.0014  0.004^c^ ± 0.0007  0.009^ab^ ± 0.0007 | | 0.009^ab^ ± 0.0009  0.006^c^ ± 0.0008  0.004^c^ ± 0.0006  0.007^b^ ± 0.0015 | 0.007^c^ ± 0.0003  0.009^b^ ± 0.0008  0.003^c^ ± 0.0005  0.009^a^ ± 0.0003 | | | 0.011^a^ ± 0.0004  0.009^b^ ± 0.0014  0.008^ab^ ± 0.0010  0.008^ab^ ± 0.0009 |
| R-PROL | Inowrocław  Ciechocinek  Saltzgraben  Soltquelle | 4.37^cd^ ± 0.049  3.80^b^ ± 0.494  2.43^c^ ± 0.045  2.84^cd^ ± 0.029 | | 4.48^cd^ ± 0.140  2.54^de^ ± 0.064  2.83^b^ ± 0.056  2.56^d^ ± 0.073 | 4.10^cd^ ± 0.129  2.49^e^ ± 0.077  2.55^bc^ ± 0.069  5.89^a^ ± 0.104 | | 5.74^a^ ± 0.144  2.83^cd^ ± 0.078  2.51^bc^ ± 0.064  4.80^b^ ± 0.225 | 4.01^d^ ± 0.125  3.03^c^ ± 0.155  2.82^b^ ± 0.345  3.02^c^ ± 0.110 | | | 5.43^ab^ ± 0.076  4.44^a^ ± 0.077  3.57^a^ ± 0.023  3.03^c^ ± 0.114 |

Abbreviations: S and R stands for shoots and roots respectively: POD - guaiacol peroxidase, CAT - catalase, SOD - superoxide dismutase, H_2_O_2_ - hydrogen peroxide, MDA - malondialdehyde, PROL - proline. Average values with standard deviation (SD) are given n=3. Two-way ANOVA with the Holm-Sidak method main effects between the tested salt concentrations in each population of *Salicornia europaea* are marked by letters per row and are significantly different at p-value < 0.05.
